# Stocking pattern for anti-malarial medications among proprietary patent medicine vendors in Akinyele Local Government Area, Ibadan, Nigeria

**DOI:** 10.1186/s12936-020-03350-1

**Published:** 2020-08-03

**Authors:** Mary Y. Kodaolu, Adeniyi F. Fagbamigbe, IkeOluwapo O. Ajayi

**Affiliations:** Department of Epidemiology and Medical Statistics, Faculty of Public Health, College of Medicine, University of Ibadan, Ibadan, Nigeria

**Keywords:** Proprietary patent medicine vendors, Recommended anti-malarial, Artemisinin-based combination therapy (ACT), Stocking, Knowledge, Perception

## Abstract

**Background:**

Policymakers have recognized that proprietary patent medicine vendors (PPMVs) can provide an opportunity for effective scaling up of artemisinin-based combination therapy (ACT) since they constitute a major source of malaria treatment in Nigeria. This study was designed to determine the stocking pattern for anti-malarial medications, knowledge of the recommended anti-malarial medicine among PPMVs in Akinyele Local Government Area (LGA) of Oyo State, Nigeria and their perception on ways to improve PPMV adherence to stocking ACT medicines.

**Methods:**

A cross-sectional survey was conducted among 320 PPMVs using a mixed method of data collection. Survey respondents were consecutively selected as a complete listing of all the PPMVs was not available. A pretested interviewer-administered questionnaire was used to collect quantitative data and two focus group discussions (FGD) were conducted among PPMVs using a pretested FGD guide.

**Results:**

Most PPMVs stocked artemether-lumefantrine (90.9%), dihydroartemisinin-piperaquine (5.3%) and artesunate-amodiaquine (2.8%). Drugs contrary to the policy, which included sulfadoxine-pyrimethamine, chloroquine, quinine, halofantrine, artesunate, and artemether were stocked by 93.8, 22.8, 0.6, 1.3, 6.6, and 7.8% of the PPMVs, respectively. Most PPMVs (96.3%) had good knowledge of artemether-lumefantrine as the first-line treatment for malaria and 2.8% had good knowledge of artesunate-amodiaquine as the alternate treatment for malaria. The major factors influencing stocking decision were government recommendations (41.3%) and consumer demand (40.30%).

**Conclusion:**

Stocking of artemisinin-based combinations was high among PPMVs, although they also stocked and dispensed other anti-malarial drugs and this has serious implications for drug resistance development. The PPMVs had considerable knowledge of the recommended treatment for uncomplicated malaria and stocking decisions were overwhelmingly driven by consumer demand. However, there is a need for more enlightenment on discontinuation of government-banned anti-malarial drugs.

## Background

According to the World Health Organization, an estimated 216 million cases of malaria occurred globally and the WHO African Region continues to account for about 90% of all malaria cases and malaria-related deaths worldwide, with about 25% occurring in Nigeria as of 2016 [1]. Many people with malaria do not attend health facilities and choose self-medication, using drugs bought from private sectors such as proprietary patent medicine vendors (PPMVs) because they are readily accessible [2]. PPMVs are a particularly important access point for basic health services among rural and poor households [3]. Even though treatment may be inappropriate and practice may not be consistent with national treatment guidelines, most patients often prefer private sector, such as PPMVs, due to their accessibility, availability and lower cost of treatment [4]. Furthermore, less effective medicines are widely available and considerably cheaper in private-for-profit outlets, such as PPMVs, where patients frequently seek malaria treatment [5].

Although artemisinin-based combination therapy (ACT) has been adopted for first-line treatment of uncomplicated malaria since 2005 in Nigeria, evidence abounds on the improper use of anti-malarial drugs, such as the use of monotherapy and other less effective anti-malarial drugs, as well as inappropriate use of ACT [6]. The use of monotherapy and ineffective drugs remains significantly high due mainly to the prevalence of self-medication as the predominant mode of malaria treatment in the sector, in addition to poor treatment practices of providers [7]. Inadequate and poor knowledge and practices in the use of artemisinin-based combinations, which is the government-recommended anti-malarial medicine, increases morbidity and mortality, undermines therapeutic efficacy and promotes the emergence and spread of drug-resistant malaria parasites.

Although PPMVs provide services for a wide variety of health needs, such as malaria, diarrhoea, respiratory infections, tuberculosis, reproductive health, common cold and cough, the quality of their services is low [8]. Generally, PPMVs have low health knowledge and poor health treatment practices, stock poor-quality medicines [9] and sub-standard formulations [10] as well as stock medicines and commodities that are prohibited for sale [11]. In the past decade, national and international efforts to combat malaria have intensified; however, problems with availability, supply and distribution of safe and effective anti-malarials continue to exist [12]. The percentage of PPMVs who stocked essential health commodities varied widely by commodity type, as well as by rural and urban location; Oyo State is one of the poor states in terms of stocking ACT, which is the recommended anti-malarial medication [13] and there are serious implications of using anti-malarials other than ACT such as artemisinin or other monotherapy for public health. Hence, this study was designed to determine the stocking pattern for anti-malarial medications, knowledge of the recommended anti-malarial medicine among PPMVs, factors that influence the types of anti-malarial medications, PPMVs’ stock and PPMVs’ perception on ways to improve adherence to stocking artemisinin-based combinations.

## Methods

### Study area

**Fig. 1:**
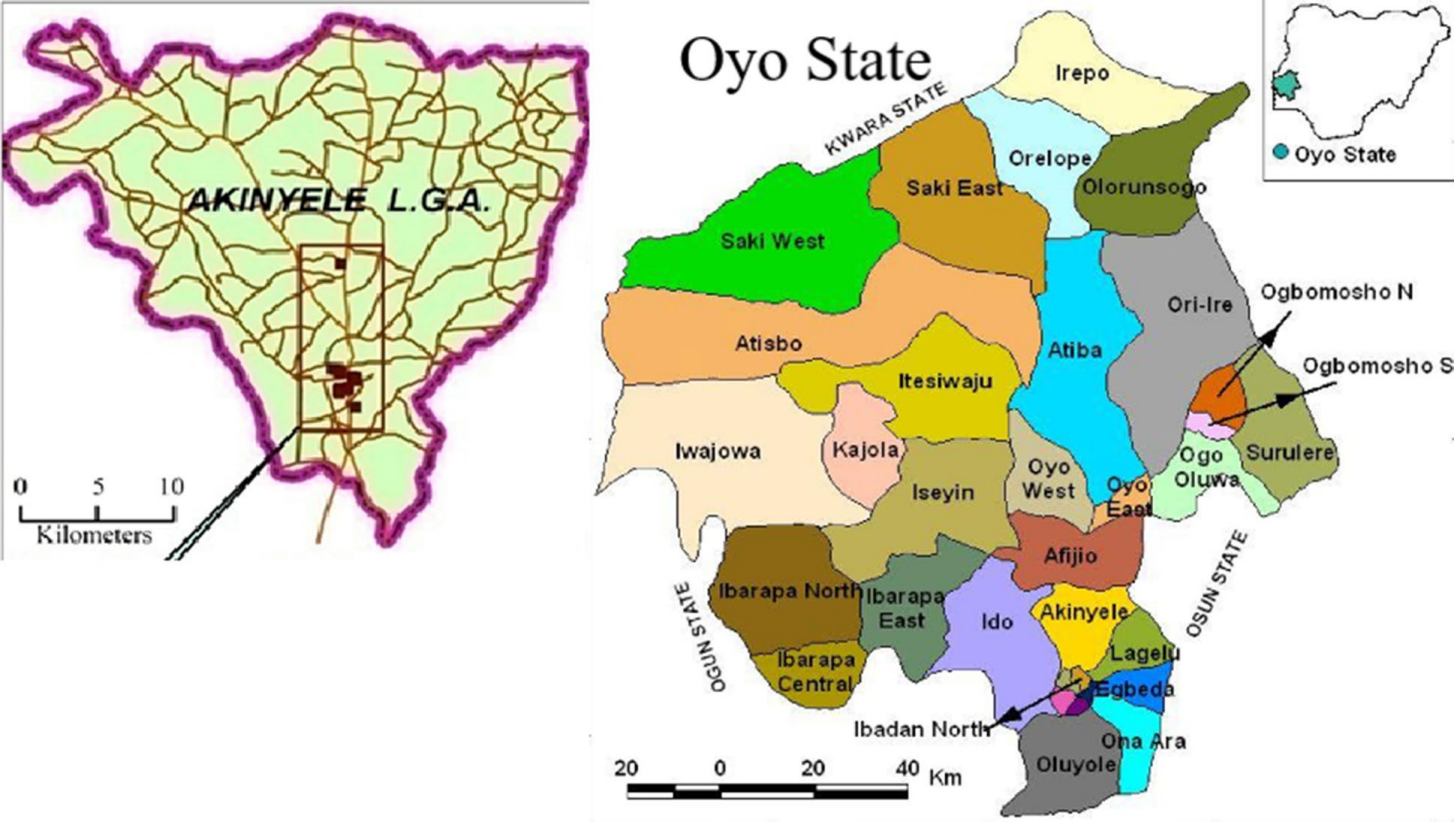
Map showing the location of Akinyele Local Government Area within Oyo State of Nigeria

This study was conducted in Akinyele LGA in Ibadan, Oyo State, Nigeria across the urban and rural locations. Ibadan is the capital of Oyo State and Ibadan metropolis has 273 public health facilities and 752 private health facilities [14]. Of the 273 public health facilities and 752 private health facilities, Akinyele LGA has 31 public health facilities and 25 private health facilities. The public health facilities are made of primary health care facilities, secondary and tertiary health facilities while the private health facilities are made of private hospitals, clinics, and maternity centres [14]. Akinyele LGA is one of the eleven local governments that make up Ibadan metropolis whose headquarter is at Moniya. Akinyele LGA is endemic for malaria, similarly to other parts of Oyo State, and home management of malaria using drugs bought from PPMVs is a common practice among caregivers in Nigeria including those in this study LGA (Fig. 1).

### Study design

A cross-sectional study was carried out. A mixed-method comprising quantitative and qualitative approaches was used to collect data. For quantitative, a survey was conducted and focus group discussions (FGD) was carried out for qualitative.

### Study population

All PPMVs who stocked any anti-malarial medicine within the 3 months before the survey were included in the study. The PPMVs were also the shop owners. The minimum sample size for this study was estimated using the Leslie Kish formula for single proportion assuming 95% level of confidence, the proportion of PPMVs stocking ACT at 25% [13], 5% precision and 10% for an anticipated non-response rate of the respondents gave a minimum sample size of 320.

### Sampling technique

Purposive selection of Akinyele LGA in Ibadan was made. The LGA is one of the few in Oyo State with both rural and urban settlements; the LGA was stratified into urban and rural locations. Based on availability at the time of visit, consecutive recruitment of 320 PPMVs (160 in urban and 160 in rural) was made because a complete listing of all PPMVs was not available.

### Data collection

Participants were interviewed using a semi-structured, interviewer-administered questionnaire adapted from the Affordable Medicines Facility-malaria (AMFm) multi-country, independent evaluation survey and an FGD guide to collect data. Questionnaire administration commenced after obtaining approval to conduct the study from Oyo State Ethical Review Committee and after informed consent was obtained from respondents. Information was collected on socio-demographic characteristics, types of anti-malarial medications stocked, knowledge of PPMVs on recommended anti-malarial medication, drivers of the type of anti-malarial medications stocked by PPMVs, and perceptions of PPMVs on ways to encourage stocking only recommended anti-malarial medications. Two FGDs were conducted among the PPMVs in the local language (*Yoruba*) with the aid of an FGD guide, and were conducted in secluded places. Each lasted about 45 to 60 min. A tape recorder was used to record the interviews and a notebook was used to make some jottings.

### Data analysis

For quantitative data, serial numbers were written on the copies of the questionnaire for easy entry and recall. A coding guide was developed along with the data collection tool to facilitate its analysis. Statistical Package for Social Sciences (SPSS) version 21 was used to analyse data obtained from the questionnaire. Using the coding guide, the data collected were carefully entered into the statistical software, cleaned and analysed. Demographic variables were summarized using descriptive statistics. Continuous variables such as age and years of experience as a PPMV were summarized by mean and standard deviation, categorical variables including gender, religion, marital status among others by frequency and proportions. Association between two categorical variables was tested using the Chi square test. Respondents’ knowledge of the recommended anti-malarial medication was measured on a 6-items and 6-point knowledge scale extracted from relevant literature on knowledge of recommended diagnosis and treatment. The total score for each respondent was obtained and the mean of the total respondent’s scores was generated. Respondents who scored below the mean score were considered to have poor knowledge of the recommended anti-malarial medication while those who scored higher than or equal to the mean value were considered knowledgeable about the recommended anti-malarial medication. Chi square and binary logistic regression were carried out to identify factors influencing the type of anti-malarial medications stocked and predictors of stocking of ACT medicines. The most important factors identified to influence stocking decision were summarized and reported by proportions and presented using a bar chart.

For qualitative data, the recorded discussions were transcribed verbatim following each session by transcribers and then translated to English by two native speakers with good command of both languages. For quality assurance purposes, the scripts were compared with the written notes for completeness and accuracy. Further, each script was checked against the audiotape by an independent reviewer. To verify the quality of translations, tapes were doubly transcribed, after which both scripts were checked for similarity and where differences existed, these were reconciled by the transcribers. Coding of transcripts was done based on emergent themes. Four themes emerged from the study and included knowledge of PPMV on government recommended anti-malarial, factors influencing PPMVs’ stocking decision, diagnostic testing and perceptions of PPMVs on ways they can be encouraged to stock only the recommended anti-malarial medications.

## Results

### Socio-demographic characteristics of respondents

A total of 320 PPMVs in urban and rural areas of Akinyele LGA, Ibadan took part in the quantitative aspect of the study. The mean age of PPMVs was 31.6 ± 5.7 years. The majority, 309 (96.6%) of the respondents were female, 297 (92.8%) were married and about two-thirds, 209 (65.3%) were of the Islamic faith. Most, 265 (82.8%) of the respondents had secondary education as their highest level of education and 306 (95.6%) of the respondents do not have a health-related qualification. The mean year of experience as a PPMV was 6.9 ± 4.7 years and the maximum number of staff working in an outlet was three (Table 1).

**Table 1:** Socio-demographic characteristics of respondents

Variables	Frequency (n)	Percentage (%)
Age group (years)		
20–24	25	7.8
25–29	98	30.6
30–34	106	33.1
35–39	63	19.7
≥ 40	28	8.8
Gender		
Female	309	96.6
Male	11	3.4
Location of outlet		
Urban	160	50.0
Rural	160	50.0
Marital status		
Married	297	92.8
Single	22	6.9
Widowed	1	0.3
Religion		
Islam	209	65.3
Christianity	110	34.4
Traditional	1	0.3
Highest level of education		
Primary	3	0.9
Secondary	265	82.8
Tertiary	52	16.3
Health-related qualification		
Not qualified	306	95.6
Qualified	14	4.4
Health qualification acquired		
Junior community health worker	7	50.0
Nurse/midwife	3	21.4
Not specified	3	21.4
Community health worker	1	7.2
Years of experience as PPMV (years)		
1–5	150	46.9
6–10	115	35.9
> 10	55	17.2
Training on malaria treatment		
Received training	144	45.0
Not received training	176	55.0
Number of staff in a PPMV outlet		
One	269	84.1
Two	38	11.9
Three	13	4.0
Other jobs aside PPMV		
No other job	274	85.6
Have other jobs	46	14.4

### Types of anti-malarial medications stocked by PPMVs

- Artemisinin-based combinations: The majority of the PPMVs, 291 (90.9%) had artemether lumefantrine (AL) in stock, 9 (2.8%) of the PPMVs had artesunate-amodiaquine (ASAQ) in stock, while 17 (5.3%) PPMV had dihydroartemisinin-piperaquine (DHAPQ) in stock.
- Artemisinin monotherapy (AMT): 22 (6.6%) PPMVs had artesunate in stock, while 25 (7.8%) stocked artemether.
- Non-artemisinin monotherapy (NAMT): most PPMVs, 300 (93.8%), had sulfadoxine-pyrimethamine (SP) in stock, 73 (22.8%) stocked chloroquine, 2 (0.6%) stocked quinine, while 4 (1.3%) stocked halofantrine. By location of PPMVs, the proportions of ASAQ, artemether and chloroquine in stock were significantly different for urban and rural locations (Table 2).

**Table 2:** Types of anti-malarial medication stocked by location

Antimalarial	Stock status	Urban n (%)	Rural n (%)	Total	*X*^2^	p-value
ACT						
Artemether	Out-of-stock	15 (9.4)	14 (8.8)	29	0.038	0.846
Lumefantrine	In-stock	145 (90.6)	146 (91.2)	291		
Artesunate	Out-of-stock	159 (99.4)	152 (95.0)	311	5.602	0.018*
Amodiaquine	In-stock	1 (0.6)	8 (5.0)	9		
Dihydroartemisinin	Out-of-stock	151 (94.4)	152 (95.0)	303	0.062	0.803
Piperaquine	In-stock	9 (5.6)	8 (5.0)	17		
AMT						
Artesunate	Out-of-stock	152 (95.0)	147 (91.9)	299	1.274	0.259
	In-stock	8 (6.0)	13 (8.1)	21		
Artemether	Out-of-stock	156 (97.5)	139 (86.9	295	12.540	˂ 0.01*
	In-stock	4 (2.5)	21 (13.1)	25		
NAMT						
Sulfadoxine	Out-of-stock	8 (5.0)	12 (7.5)	20	0.853	0.356
Pyrimethamine	In-stock	152 (95.0)	148 (92.5)	300		
Chloroquine	Out-of-stock	146 (91.2)	101 (63.1)	247	35.938	˂ 0.01*
	In-stock	14 (8.8)	59 (36.9)	73		
Quinine	Out-of-stock	158 (98.8)	160 (100.0)	318	2.013	0.156
	In-stock	2 (1.2)	0	2		
Halofantrine	Out-of-stock	159 (99.4)	157 (98.1)	316	1.013	0.314
	In-stock	1 (0.6)	3 (1.9)	4		

*ACT* artemisinin combination therapy, *AMT* artemisinin monotherapy, *NAMT* non-artemisinin monotherapy

* Significant p-value

### Frequency of stocking anti-malarial medications

The majority, 155 (48.4%) of the PPMVs stocked the most sold anti-malarial ≤ 5 times monthly, 157 (49.0%) of the PPMVs stocked any anti-malarial medication ≤ 5 times monthly, while 187 (58.4%) stocked ACT which is the recommended anti-malarial medication ≤ 5 times in a month (Table 3).

**Table 3:** Frequency of stocking anti-malarial medication by PPMVs

Variable	Monthly	Frequency	Percentage (%)
Frequency of stocking most sold anti-malarial medication	≤ 5 times	150	47.6
	6–10 times	148	47.0
	> 10 times	17	5.4
Frequency of stocking any anti-malarial medication	≤ 5 times	152	48.3
	6–10 times	143	45.4
	> 10 times	20	6.3
Frequency of stocking ACT	≤ 5 times	184	58.0
	6–10 times	118	37.2
	> 10 times	15	4.7

### Sources of and storage of anti-malarial drugs by the PPMVs

Almost all PPMVs, 319 (99.7%) purchased anti-malarials from wholesalers, 9 (2.8%) purchased from drug sales representatives, 4 (1.3%) purchased from manufacturers and 1 (0.3%) purchased from the local market. The majority, 307 (95.9%) of the PPMVs placed an order for anti-malarial by going to their supplier, while 13 (4.1%) placed an order for anti-malarials via telephone. The majority, 318 (99.4%) of the PPMVs stored medicines in a dry area in their stores and 299 (93.4%) of the stores’ anti-malarials were protected from direct sunlight. Only 1 (0.3%) store kept anti-malarials on the floor. The majority, 314 (98.1%) of the stores’ anti-malarials were kept out of reach and sight of children. Most of the stores, 302 (94.4%) had tidy and washable floors.

### Knowledge of PPMVs on the recommended anti-malarial medication

Some of the PPMVs in the focus group discussion correctly stated AL to be the government recommended anti-malarial medicines for treating uncomplicated malaria. Some also mentioned SP and Chloroquine. This was expressed as follows:

*“Part of the government recommended anti-malarial medicines for sales are the likes of Lumartem and Combisunate*^1^” (Male discussant, urban area)

*“Government recommended anti-malarial medicines are Amalar, Lifloxine and sulfadoxine for single-dose then we have the three doses which is ACT such as Lumartem, Somal and Shalartem*^2^” (Male discussant, rural area)

*“The anti-malarial medicines government recommended that we sell are ACT and we have categories of ACT which are by one, by two, by three and by four. An example is Lonart.*^3^*We also have chloroquine and sulfadoxine pyrimethamine recommended by the government for sales”* (Female discussant, urban area)

A participant mentioned artemether to be the recommended anti-malarial medicine for treating uncomplicated malaria as quoted below:

*“The government recommended anti*-*malarial medicines are artemether drugs for both adult and children since chloroquine has been banned from usage”* (Female discussant, rural area)

Some participants mentioned herbal mixture to be the recommended anti-malarial medicines to which most of the participants nodded in agreement. This was stated as follows:

*“We have herbal products recommended by the government such as Champion and Easy cure. These herbal products are even used beyond Nigeria”* (Male discussant, rural area)

*“In addition to what has been said, there are also herbal mixtures like briskly herbal mixture, easy cure herbal mixture. They are also available in different kinds and are recommended by the government”* (Male discussant, urban area)

The majority, 308 (96.3%) of the PPMVs correctly reported AL as the first-line treatment for malaria and 9 (2.8%) correctly reported ASAQ as the alternate treatment for malaria (Table 4). The mean Knowledge Score (KS) on recommended anti-malarial medication was 3.0 ± 1.0 with minimum and maximum scores of 1.0 and 6.0 respectively. Most, 204 (63.8%) of the respondents had good knowledge (Table 5).

**Table 4:** Knowledge of PPMVs on the recommended anti-malarial medication

Knowledge variables	Responses	Frequency	Percentage (%)
Knowledge of AL as first-line treatment for uncomplicated malaria	Correct knowledge	308	96.3
	Incorrect knowledge	6	1.9
	Don’t know	6	1.9
Knowledge of ASAQ as alternate treatment for uncomplicated malaria	Correct knowledge	9	2.8
	Incorrect knowledge	279	87.2
	Don’t know	32	10.0
Knowledge of national recommended guideline for malaria diagnosis	Correct knowledge	8	2.5
	Incorrect knowledge	53	16.6
	Don’t know	259	80.9
Microscopy test should be done before dispensing anti-malarial	No	210	78.4
	Yes	58	21.6
Rapid diagnostic test should be done before dispensing anti-malarial	No	74	27.6
	Yes	194	72.4
Test should be done using a thermometer before dispensing anti-malarial	No	261	97.4
	Yes	7	2.6

**Table 5:** Knowledge score of PPMVs on recommended anti-malarial medications

Knowledge score (KS)	Frequency	Percentage (%)
Good knowledge (KS ≥ 3)	204	63.8
Poor knowledge (KS˂3)	116	36.3
Total	320	100

**Fig. 2:**
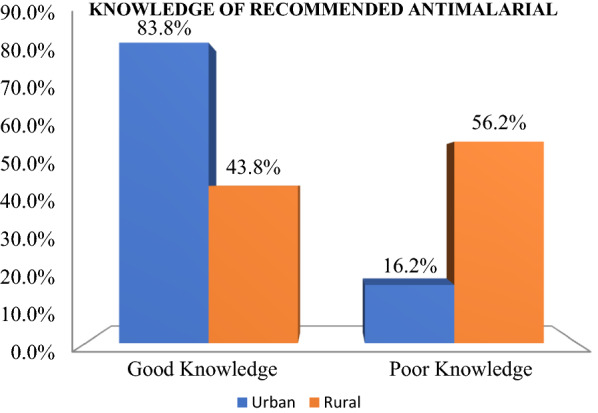
Respondents’ knowledge of recommended anti-malarial medication

The various sources from which PPMVs acquired information on the recommended anti-malarial medications included association meeting 293 (91.6%), media (TV, radio, newspaper) 105 (32.8%), others 35 (11.1%) which included friends, colleagues, family members, customers and private hospitals, sales representative 17 (5.3%), internet 12 (3.8%), organized PPMVs training 9 (2.8%), government agencies 5 (1.6%) and seminars and product launch 4 (1.3%). Respondents in the urban locations, 134 (83.8%), were more knowledgeable than their rural counterparts, 70 (43.8%) (Fig. 2).

### Factors influencing the types of anti-malarial medications stocked

In the FGD, most PPMVs identified consumer demand as a major factor influencing their stocking decision. Other factors mentioned included profit-making, less costly medicines and affordability. This was expressed this way:

*“Although profit*-*making is minimal, it is majorly based on what my customers demand the most/consumer demand”* (Male discussant, rural area)

*“Firstly, the nature of our work is health*-*related and it has come to our notice that malaria is common and occurs frequently, therefore, anti*-*malarial medicines is a must*-*have in stock since our customers ask for it and for*-*profit”* (Male discussant, urban area)

*“Another factor we consider in the stocking of anti*-*malarial medicines is profit*-*making. Since malaria is common in the community, therefore customers will definitely request for anti*-*malarial medicines”* (Female discussant, rural area)

*“Since we are the closest to the community members, therefore, we must have anti*-*malarial medicines in stock because customers will demand it because if patients are to go to the hospital, they would first be required to do test which might be costly aside from the drugs they will buy but when they come to us we have all kinds of anti*-*malarial medicines with varying prices which customers can choose from based on the money they have at hand”* (Female discussant, urban area)

Some of the participants mentioned government recommendation as a factor that influences their stocking decision:

*“Government recommendation is also a factor since the likes of chloroquine have been banned from consumption in malaria treatment”* (Female discussant, rural area)

*“In addition, government recommendations and NAFDAC approval are also factors influencing our stocking decision. The government should ensure that these NAFDAC approved anti*-*malarial medicines are available in large quantities at the hospitals and pharmaceutical companies”* (Male discussant, rural area)

Majority 285 (89.1%) of the respondents considered consumer demand and 254 (79.4%) mentioned government recommendation as factors they consider before stocking any anti-malarial medication (Table 6). In the survey, factors influencing PPMVs’ decision not to stock AL included when PPMV is temporarily out of stock for AL 311 (97.5%), AL is too costly 128 (40.1%), unavailability of AL at the suppliers 66 (20.7%), customers do not ask for AL 10 (3.1%), AL is not profitable 3 (0.9%), AL does not work well 1 (0.3%) and when PPMV does not know about AL 1 (0.3%); while factors influencing PPMVs’ decision, not to stock artesunate-amodiaquine (ASAQ) included ASAQ has too many side effects 299 (93.4%), customers do not ask for ASAQ 129 (40.3%), when PPMV is temporarily out of stock for ASAQ 11 (3.4%), ASAQ is too costly 7 (2.2%), PPMV does not know about ASAQ 5 (1.6%), ASAQ is not profitable 3 (0.9%), unavailability of ASAQ at the suppliers 3 (0.9%) and when an outlet is not allowed to sell ASAQ 2 (0.6%).

**Table 6:** Factors influencing the types of anti-malarial medications stocked by PPMVs

Factors*	Frequency	Percentage (%)
Consumer demand	285	89.1
Recommended by the government	254	79.4
Lowest priced	102	31.9
Prescribed most often by doctor	94	29.4
Amount of cash available	53	16.6
Expiration dates	39	12.2
Levels of remaining stock	23	7.2
Drug company/sales rep influence	9	2.8
Most profitable	7	2.2
Brand reputation	7	2.2
Easily available	6	1.9
Provider incentives	1	0.3

* Multiple responses

### Predictors of ACT stocking

#### Factors associated with stocking artemether-lumefantrine (AL)

Proprietary patent medicine vendors with tertiary education and PPMVs who had less than tertiary education was equally likely to stock AL; The odds of stocking AL among the PPMVs with over 5 years’ experience were about 2 times higher than among PPMVs with less than 5 years’ experience (Table 7).

**Table 7:** Predictors of stocking of AL-ACT by PPMVs

Variables	Artemether (AL) in yes n (%)	Lumefantrine stock no n (%)	*X*^*2*^ (p-value)	OR (95% CI)
Level of education				
Tertiary (ref)	239 (89.2%)	29 (10.8%)		
Below tertiary	52 (100.0%)	0 (0.0%)	6.19 (0.013)*	1
Years of experience as a PPMV				
≤ 5 years (ref)	131 (87.9%)	18 (12.1%)		
More than 5 years	159 (93.5%)	11 (6.5%)	3.02 (0.082)	1.99 (0.91–4.35)
PPMV knew AL is the government recommended anti-malarial for uncomplicated malaria				
Correct knowledge (ref)	282 (91.6%)	26 (8.4%)		
Incorrect knowledge	9 (75.0%)	3 (25.0%)	3.84 (0.050)	0.28 (0.07–1.09)

*Ref* reference category

* Significant p-value

#### Factors associated with stocking artesunate-amodiaquine (ASAQ)

The odds of stocking ASAQ among the PPMVs in rural area were about 8 times higher than among their urban counterparts. The odds of stocking ASAQ among the PPMVs who had received training on malaria treatment in the last 12 months was over 4 times higher than among those who have not received training. The odds of stocking ASAQ among the PPMVs who had incorrect knowledge of ASAQ as the alternate recommended anti-malarial for treatment of uncomplicated malaria were about 92% lower than among PPMVs with correct knowledge (Table 8).

**Table 8:** Predictors of stocking of ASAQ-ACT by PPMVs

Variables	Artesunate (ASAQ) yes n (%)	Amodiaquine in stock no n (%)	*X*^*2*^ (p-value)	OR (95% CI)
Outlet type				
Urban (ref)	1 (0.6)	159 (99.4)		
Rural	8 (5.0)	152 (95.0)	5.60 (0.018) *	8.37 (1.03–67.71)
Training on malaria treatment in the last 12 months				
Not received training (ref)	2 (1.1)	174 (98.9)		
Received training	7 (4.9)	137 (95.1)	4.02 (0.045)	4.45 (0.91–21.74)
PPMV knew ASAQ is the alternate anti-malarial recommended by the government for uncomplicated malaria				
Correct knowledge (ref)	2 (22.2)	7 (77.8)		
Incorrect knowledge	7 (2.3)	304 (97.7)	12.76 (0.001) *	0.08 (0.01–0.46)
Government agencies as source of information on government recommended anti-malarial medication				
No (ref)	8 (2.5)	307 (97.5)		
Yes	1 (20.0)	4 (80.0)	5.49 (0.019) *	9.59 (0.96–95.78)
PPMV knew the national recommended guideline for malaria diagnosis				
Incorrect knowledge (ref)	8 (2.6)	304 (97.4)		
Correct knowledge	1 (12.5)	7 (87.5)	2.82 (0.093)	5.43 (0.60––49.47)

*Ref* reference category

* Significant p-value

#### Factors associated with stocking dihydroartemisinin-piperaquine (DHAPQ)

The odds of stocking DHAPQ among the PPMVs with health-related qualification were about 5 times higher than among PPMVs without health-related qualification. The odds of stocking DHAPQ among the PPMVs who obtained information on the recommended anti-malarial from sales representatives were about 4 times higher than among PPMVs who did not obtain information from sales representatives. The odds of stocking DHAPQ among the PPMVs who had the correct knowledge of the national recommended guideline on malaria diagnosis were about 6 times higher than among PPMVs with incorrect knowledge (Table 9).

**Table 9:** Predictors of stocking of DHAPQ-ACT by PPMVs

Variables	Dihydroartemisinin (DHAPQ) in yes n (%)	Piperaquine stock no n (%)	*X*^*2*^ (p-value)	OR (95% CI)
Health-related qualification				
No health qualification (ref)	14 (4.6)	292 (95.4)		
Has health qualification	3 (21.4)	11 (78.6)	7.56 (0.006)*	5.69 (1.42–22.72)
Manufacturer as the source of purchase of anti-malarial				
No (ref)	16 (5.1)	300 (94.9)		
Yes	1 (25.0)	3 (75.0)	3.12 (0.077)	6.25 (0.62–63.49)
Sales representatives as a source of information on government recommended anti-malarial medication				
No (ref)	14 (4.6)	289 (95.4)		
Yes	3 (17.6)	14 (82.4)	5.43 (0.020)*	4.42 (1.14–17.19)
PPMV knew the national recommended guideline for malaria diagnosis				
Incorrect knowledge (ref)	15 (4.8)	297 (95.2)		
Correct knowledge	2 (25.0)	6 (75.0)	6.32 (0.012)*	6.60 (1.23–35.49)
PPMV used malaria diagnostic tool				
No (ref)	14 (4.7)	286 (95.3)		
Yes	3 (15.0)	17 (85.0)	3.98 (0.046)	3.61 (0.94–13.76)

*Ref* reference category

* Significant p-value

#### Perception on ways to improve adherence to stocking recommended anti-malarial medicines by PPMVs

In the focus group discussion, PPMVs reported consistent availability and price reduction of ACT as means of encouraging stocking of the recommended anti-malarial medicines. This was supported by the following quotes:

*“There should be consistent availability of the anti*-*malarial medicines at the suppliers/where we buy them so these anti*-*malarial medicines will always be available when demanded by our customers who are already familiar with these anti*-*malarial medicines. For example, Lumartem is too expensive. Therefore, these recommended anti*-*malarial medicines should be made less costly such that we too can gain profit no matter how little”* (Female discussant, rural area)

*“The way I know we can be encouraged in stocking the recommended anti*-*malarial medicines is by helping us to reduce the price of ACT such as Lumartem. They are too costly but if the price is reduced, we will be able to stock more ACT than we do now”* (Female discussant, urban area)

Other means of encouragement to stocking the recommended anti-malarial medicines mentioned were continuous education, granting of loans and use of social media/website to publicize any information associated with PPMVs. These were expressed this way:

*“One of the ways we can be encouraged is if there is continuing education for us. We should be trained so that we can be more exposed to the anti*-*malarial medicines recommended by the government and from there the same knowledge can be passed on to other members”* (Female discussant, urban area)

*“If the government can give us loans and the pharmaceutical companies give us these recommended anti*-*malarial medicines on credit, then we would be able to stock these medicines the more”* (Male discussant, rural area)

*“In addition to what has been said, the government should try as much as possible to give us adequate training for us to know the recommended drugs. Old drugs since the days of 1970s were very effective like quinine, if the government can introduce these drugs back instead of producing new drugs which people are not familiar with; even those producing these drugs will no longer sell us fake drugs. The government should also try to return the old ways/system by which these drugs circulate”* (Male discussant, urban area)

*“Technology is all over now, there is nothing stopping the government from having a website through which the government can make us aware of banned drugs because part of the issues we are having with the police is that we stock some drugs having NAFDAC registration number but they have been banned even with the NAFDAC number unknown to us. There is insufficient publicity for us to know and differentiate these banned drugs from the recommended drugs, therefore, the government should make use of social media and a special website to pass any information across to us to increase publicity”* (Male discussant, urban area)

## Discussion

This study assessed and determined the stocking pattern for anti-malarial medications, knowledge of the recommended anti-malarial medicine and factors associated to stocking patterns among PPMVs in Akinyele LGA of Oyo State of Nigeria. The study showed that nearly all PPMVs did not have any health-related qualification while half of those who had health-related qualification were junior community health workers (JCHEW). This was at variance to the outcome of a study among drug store owners in southwestern Uganda, which reported that the majority who had a health-related qualification were nurses/midwives [15]. Nearly all PPMVs who received training on malaria treatment were trained by the Nigerian Association of Patent and Proprietary Medicine Dealers (NAPPMED). This could be because the PPMVs are more often registered with their professional association, with 92% registered with NAPPMED and 15% registered with PCN, an organization, which they were required to register with [13]. The maximum number of staff working in a PPMV store was three. This corroborates with findings of Shah et al. among drug vendors where the majority of drug stores had between 2 and 3 staff [16].

The majority of the respondents stocked ACT. This is contrary to the outcome of a study by Adibe et al. among PPMVs in Enugu State where ACT was the least stocked drug [17]. The ACT-stocking level found in the current study was higher compared with the findings of Oladepo et al. among PPMVs in 3 Nigerian states [18] and Liu et al. among PPMVs in 16 states of Nigeria where it was reported that Oyo State has poor practice of ACT stocking [13]. The significant improvement in ACT-stocking in Oyo State from the time of the study by Oladepo et al. and the current study could be due to increasing knowledge and awareness of the policy change from chloroquine to ACT. Over the past 14 years ACT was adopted through educational programmes, increased PPMV access to ACT supply chains and increased ACT distribution.

Artemisinin lumefantrine which is the policy first line of treatment in Nigeria was the most commonly stocked ACT. Of the PPMVs who stocked ACTs, 90.9% of PPMVs stocked Artemisinin lumefantrine, 5.3% of PPMVs stocked Dihydroartemisinin-piperaquine while only 2.8% of PPMVs stocked Artesunate-amodiaquine. This finding is very similar to the result obtained in previous studies [7, 19]. The stocking of ASAQ was poor. This finding is similar to results obtained by Kioko et al. in a cross-sectional study conducted among private-sector retail drug outlets inclusive of PPMVs in Kenya [19] but contrary to findings of Mangham et al. in Southeastern Nigeria which reported that 78.8% of medicine retailers including PPMVs had ASAQ in stock [6]. The relatively low stocking of ASAQ by PPMVs could be attributed to the public perception that ASAQ has too many side effects and intolerable which in turn influences consumer demand. Hence, side effects reported by customers could affect stocking of ASAQ by PPMVs because customers would not ask for it.

Although many PPMVs stocked ACT, a majority stocked less expensive non-artemisinin monotherapy such as SP also known as Fansidar (93.8%), chloroquine (22.8%), quinine (0.6%), halofantrine (1.3%) and artemisinin monotherapy such as artesunate (6.6%), artemether (7.8%) which has serious implications for the development of drug resistance. These findings are similar to the result obtained by Oladepo et al. [18] in a study conducted among PPMVs in three Nigerian states inclusive of Oyo State, Berendes et al. [2] in a study done among PPMVs in Jigawa State, Nigeria using lot quality assurance sampling (LQAS), and Liu et al. in a study done on the landscape of PPMVs in 16 states of Nigeria [13].

The stocking frequency was high with PPMVs reporting several orders per month as similarly documented by Palafox et al. in a qualitative assessment of the private sector anti-malarial distribution chain in Nigeria [20]. Quantification of orders was due to high demand by a customer and the amount of cash available to purchase new stock. Most PPMVs purchased anti-malarial medications mostly from wholesalers. This was attributed to the nearness of wholesalers to the PPMVs, genuine wholesalers mind what to stock and sell to maintain customers and wholesalers offer credit facility which is rarely available at the open market as similarly documented by Palafox et al. who reported credit offering as a key means by which wholesalers attracted customers [21]. This is in contrast to findings of studies by Adibe et al. and Asuquo et al. who both reported open market as the major source of purchase of anti-malarials by PPMVs and attributed it to ease of access to the open market by the PPMVs and the option of bargaining for a fair price of the drugs [17, 22]. According to Asuquo et al. open market as a major source of procurement of anti-malarial medicines has led to increasing levels of substandard drugs which are commonly seen in the open market and PPMVs shops [22].

This study revealed that a high proportion of the PPMVs stored anti-malarial medications in acceptable storage conditions as similarly documented by ACT watch Group in an outlet survey conducted in Nigeria [23]. Nearly all stores’ anti-malarials were stored in a dry area place within the stores, stored out of reach of children, protected from direct sunlight, the PPMVs’ stores had washable floors and the majority of the stores had tidy floors since anti-malarial medicines were not kept on the floor rather they were stored on shelves which varies completely from the result obtained by Shah et al. among drug stores owners in Pakistan where anti-malarial medicines were stored directly on the floor which made the stores untidy [16]. Storage of anti-malarial medications in acceptable storage conditions can be attributed to the PPMVs association, the Nigerian Association of Patent and Proprietary Medicine Dealers (NAPPMED) who occasionally check on the members’ practices to monitor their adherence to guidelines.

This study finding revealed the PPMVs level of knowledge of the recommended anti-malarial medication to be mostly good which might be as a result of increased awareness about ACTs by the government and other health agencies. This finding corroborates with a previous study by Chukwuocha et al. among PPMVs in Owerri metropolis, Imo State, Nigeria who documented that PPMVs had a high level of knowledge of the recommended anti-malarial medicine [24], although PPMVs in the urban area were more knowledgeable than their rural counterpart. Hence, the government and health agencies should focus on improving the level of awareness in the rural area.

Majority of the PPMVs were able to correctly state AL as the recommended first-line treatment for uncomplicated malaria which shows better knowledge of AL compared to the knowledge reported by ACT watch Group where 15% of drug stores owner correctly mentioned AL as the first-line treatment for uncomplicated malaria [23]. This is in variance with findings of the study by Tobin-west and Adeniji among PPMVs in rural and semi-urban communities in Rivers State who documented that 9.1% of PPMVs correctly stated ACT as the recommended first-line treatment for uncomplicated malaria [25]. This improved knowledge with an increasing year since the adoption of ACT could be attributed to educational programmes, training and wider dissemination of the malaria treatment policy change to ACT. A large proportion of the respondents were unable to correctly state ASAQ as the alternate recommended treatment for uncomplicated malaria with majority mentioning SP as the alternate recommended anti-malarial medicine after 14 years of policy change. This is in agreement with the findings of Tobin-west et al. [25] and also indicates the need for proper awareness on the alternate recommended anti-malarial medicine among PPMVs.

This study finding revealed there was poor knowledge of PPMVs on the current diagnosis and treatment guidelines for malaria, which is in variance with findings by Adibe et al. [17] where the majority of the PPMVs in Nsukka, Enugu claimed to know the current treatment guideline but in agreement with the result obtained by Berendes et al. among PPMVs in Jigawa State, Nigeria [2] and among drug shop vendors in Uganda who also reported that PPMVs had poor knowledge of the current guideline for malaria diagnosis and treatment [26]. Continuous prescription of anti-malarial medicines based solely on physical examination and symptoms was observed among PPMVs, this finding is contrary to the current WHO guideline on malaria treatment which requires laboratory confirmation of malaria to reduce the indiscriminate use of effective anti-malarial drugs such as ACT [1] but similar to findings of Asuquo et al. among PPMVs in Rivers State, Nigeria and Oladosu and Oyibo in a study on malaria case management in children presenting with fever in Lagos, Nigeria who both reported that malaria is mainly diagnosed solely on the basis of symptoms in Nigeria [22, 27]. This can lead to abuse and improper use of ACT which in turn can result in negative clinical and economic impact as well as encourage the development of drug resistance. Hence there is still the need for the PPMVs to be trained periodically on the recommended guideline for diagnosis and treatment of uncomplicated malaria. There was a report on PPMVs having poor access to the recommended treatment guideline as similarly documented in a study conducted by Mangham-Jefferies et al. [28] among drug stores owners in Nigeria and Cameroon but at variance with findings of Adibe et al. in a study conducted among PPMVs in Enugu State, Nigeria which reported 75.6% had access to the recommended treatment guideline [17].

A higher percentage of information on recommended anti-malarials was sourced from the PPMVs association meetings, the Nigerian Association of Patent and Proprietary Medicine Dealers (NAPPMED). This is most likely to affect their stocking pattern, hence the need to intensify training and empowering of PPMVs through their associations. This is contrary to an earlier report by Palafox et al. that sales representatives were the most common source of information on anti-malarials [20]. The variance between this study finding and report by Palafox et al. could be attributed to the nearness of the PPMVs association, NAPPMED to its members where they also conduct weekly meetings. Media and organized training were reported as another source of information for PPMVs which corroborates findings of Chukwuocha et al. in a cross-sectional study among PPMVs in Imo State, Nigeria [24] and radio was reported by Ajayi et al. as the source of information on AMFm-ACT [29]. Other sources included friends, colleagues, family members and customers, sales representatives and government agencies. It was reported by Adibe et al. [17] that majority of PPMVs in Enugu State, Nigeria got their information on anti-malarial drugs from colloquies, government, and pharmacists while very few mentioned seminars and product launches as sources of information as documented earlier by Palafox et al. [20].

The factors identified to influence the types of anti-malarial medication stocked by PPMVs were majorly consumer demand and government recommendation. This corroborates earlier findings by Mayora et al. [15] in a cross-sectional study among PPMVs in Uganda and Palafox et al. [20] in a cross-sectional mixed-methods study in six malaria-endemic countries among private-for-profit outlets where it was documented that consumer demand was the principal consideration when selecting anti-malarial medicines in all the six countries, Nigeria inclusive [21]. It is not surprising that the stocking pattern of PPMVs was more likely to follow customers demand and the profit motive as the law of demand and supply in economics explains consumer choice behavior with changes in price. Price and quantity demand of any good and service are inversely related to each other such that when the price of a product increases, the demand for the same product will fall; and as reported by Oladepo et al. PPMVs tend to stock a variety of medicines at varying price ranges that are popular with the public and because the public clinics often run out of free or reduced-price drugs [18]. The PPMVs were more likely to stock and supply the anti-malarial medicines their customers demand hence, there is a need for community-level re-orientation on drug use and policy information dissemination to the public to make informed choices when choosing anti-malarial.

Other factors identified in the study were drug being less costly and affordable medicines that yield profit, doctor’s prescription, sales representative influence, expiration dates, levels of stock remaining and availability problems which affected the demand of anti-malarial medications. These findings are in agreement with the outcome of a qualitative assessment of the private sector anti-malarial distribution chain in Nigeria which reported that the high price of the ACT compared to other anti-malarials and availability problems which affected demand and popularity of the less expensive anti-malarials, such as chloroquine and SP, influenced the stocking decisions of both wholesalers and retailers [20].

Sales representatives who were acknowledged by PPMVs as the sources of information on anti-malarial drugs were found to have influence over the choice of anti-malarials to stock. This is contrary to the findings of Palafox et al. [20] where sales representatives did not have influence over the choice of products to stock even though they served as the source of information, but similar to that of Mayora et al. where sales representatives from pharmaceutical companies were documented to have an influence on stocking decision [15]. Findings of this study revealed that PPMVs would not have AL in stock majorly because they were temporarily out of stock which was either due to unavailability of AL at supplier level or lack of funds to stock AL due to its high cost when compared to other anti-malarial medicines. The result of this finding is comparable with reasons why retailers would not stock ACT as documented earlier by Asuquo et al. and Palafox et al. [20, 22]. Predictors of stocking of AL and ASAQ by PPMVs in this study were found to be years of experience as a PPMV, knowledge of PPMV on the recommended anti-malarial treatment for uncomplicated malaria, training on malaria treatments in the last 12 months, government agencies as source of information on anti-malarials, knowledge of PPMVs on the recommended guideline for malaria diagnosis and PPMVs having access to the national guideline for malaria diagnosis.

Although reports showed the misconception of the combination therapy causing some adverse events [22], this study found out that PPMVs would not stock ASAQ majorly because of its numerous adverse effects such as difficulty in sleeping, nausea, abdominal pain, weakness and tiredness as reported by customers based on actual experience which resulted to customers not demanding for ASAQ even though it was reported to be effective for malaria treatment. This is similar to an earlier finding by Camara et al. that a greater proportion of patients prescribed ASAQ do experience adverse drug effects such as vomiting, fatigue, dizziness and drowsiness compared to those prescribed AL [30]. Likewise, a study comparing ASAQ and AL for the treatment of uncomplicated malaria in Congolese children under 10 years old showed that ASAQ was associated with significantly more frequent adverse events [31].

Reducing the price of ACT, continuing education and community awareness program through the media on the benefits of ACT were mentioned by the PPMVs as ways to encourage stocking of ACT. This is corroborated by a previous finding of Palafox et al. [20]. As regards the Affordable Medicines Facility for Malaria (AMFm) initiative which subsidized the price of ACT, respondents bitterly complained that the subsidy was being captured by middlemen in the distribution chain; this observation has been reported by O’Connell et al. on availability, price, market share and provider knowledge of anti-malarial medicines in public and private sector outlets including PPMVs in six malaria-endemic countries [32]. Hence, PPMVs prefers to be given these subsidized ACT directly rather than going to the wholesalers to buy at expensive prices. Many respondents pointed out that if the subsidy were to come to each PPMV instead, that would reduce the high price they currently have to charge consumers for ACT. In turn, this change could make it economically feasible for PPMVs to sell more ACT.

Proprietary patent medicine vendors expressed the need for further training to improve their current practices and improve their adherence to stocking the recommended anti-malarial medication. This is similar to findings of a qualitative study in Southeast, Nigeria which reported continuous training as health workers’ perception to improve their current practices and promote the appropriate recommended treatment for malaria [33]. Consistent availability of ACT as similarly documented earlier by Chukwuocha et al. was suggested as a way by the PPMVs to encourage their stocking of ACT [24]. Other suggestions given by PPMVs to encourage their stocking of ACT were granting of loans, use of social media and website to increase publicity and information coverage, follow up visits by regulatory bodies to ascertain PPMVs stock the recommended anti-malarial, engaging NAPPMED in training, monitoring and quality assurance activities and formulation of a single dose treatment for side effects tolerance.

### Study limitations

The limitations of this study included the lack of a standard tool for assessing the stocking pattern of PPMVs, incomplete listing of all PPMVs and some respondents may be not wanting to reveal all types of anti-malarial medications they had in stock due to fear of sanction by regulatory bodies in case they had the banned anti-malarial medications in stock. The study was conducted only in Akinyele LGA of Ibadan which limits the generalizability of the findings to the entire population of PPMVs in Nigeria. However, the study was strengthened by the involvement of a large number of PPMVs and the use of mixed methods.

## Conclusion

Although most PPMVs stocked ACT, many still stocked artemisinin monotherapy and non-artemisinin monotherapy which is a less effective anti-malarial and is discouraged by Nigeria health authorities. Continued usage of non-recommended anti-malarials will undermine the therapeutic efficacy and may promote the emergence and further spread of drug resistance to the effective ACT. PPMVs had considerable knowledge of the recommended treatment for uncomplicated malaria. This could be ascribed to awareness about ACT through the various channels pointed out by PPMVs as sources of their information about ACT. It also indicates the influence of information and proper awareness on the knowledge of PPMVs about ACT. However, there is still the need for the PPMVs to be trained periodically on the recommended guideline for diagnosis and treatment of uncomplicated malaria. Stocking decisions were overwhelmingly driven by consumer demand, hence there is a need for community-level re-orientation on drug use and policy information dissemination to the public, especially mothers so that they can make informed choices when choosing anti-malarials. The high price of ACT relative to other anti-malarials and problems with availability constrained their stocking decisions. Hence, many still stocked older, less expensive and less effective anti-malarial medicines. There should be a price reduction on ACT to make it affordable.

## Data Availability

The dataset used and analyzed during the current study is available from the corresponding author on request.

## Abbreviations

ACT: Artemisinin based combination therapy
AL: Artemether-lumefantrine
AMFm: Affordable Medicines Facility-malaria
AMT: Artemisinin monotherapy
ASAQ: Artesunate-amodiaquine
ASMQ: Artesunate mefloquine
ASSP: Artesunate-sulfadoxine-pyrimethamine
AQ: Amodiaquine
CQ: Chloroquine
DHAPQ: Dihydroartemisinin-piperaquine
FGD: Focus Group Discussion
FMOH: Federal Ministry of Health
GFATM: Global Funds to Fight AIDS, Tuberculosis and Malaria
JCHEW: Junior Community Health Workers
LGA: Local Government Area
LSHTM: London School of Hygiene and Tropical Medicine
NAFDAC: National Agency for Food & Drug Administration and Control
NAPPMED: Nigerian Association of Patent and Proprietary Medicine Dealers
NMCP: National Malaria Control Programme
PCN: Pharmacists Council of Nigeria
PPMVs: Proprietary patent medicine vendors
PSI: Population Services International
RDT: Rapid diagnostic test
SFH: Society for Family Health
SP: Sulfadoxine pyrimethamine
SPSS: Statistical Package for Social Sciences
SSA: Sub Saharan Africa
WHO: World Health Organization

## Footnotes

1 Generic name of Combisunate is Artemether Lumefantrine.

2 Generic name of Lumartem, Somal and Shalartem is Artemether Lumefantrine.

3 Generic name of Lonart is Artemether Lumefantrine.
